# Shooting through time: new insights from transcriptomic data

**DOI:** 10.1016/j.tplants.2015.06.003

**Published:** 2015-08

**Authors:** C. Jill Harrison

**Affiliations:** Department of Plant Sciences, University of Cambridge, Downing Street, Cambridge, CB2 3EA, UK

## Abstract

Plant evo-devo research aims to identify the nature of genetic change underpinning the evolution of diverse plant forms. A transcriptomic study comparing gene expression profiles in the meristematic shoot tips of three distantly related vascular plants suggests that different genes were recruited to regulate similar meristematic processes during evolution.

The conquest of land by plants was one of the most significant events in our planet's history, and the radiation of diverse plant forms was underpinned by a series of ancient innovations in sporophytic shoot architecture. Whilst living bryophyte representatives of the earliest land plants have a single sporophytic axis that terminates growth by forming a reproductive sporangium (Figure 1A), todays dominant vascular plant flora has shoots, branches and leaves under every variety of form and function (Figure 1A, B).

The elaboration of these basic organ systems in vascular plants began around 400–450 million years ago [1], and the morphological distance between living bryophytes and vascular plants is wide. However, ancient fossils deriving from the colonisation of land show intermediary forms that cast light on the sequence of architectural change during evolution.

For instance, the non-vascular fossil *Partitatheca* has a branching sporophytic axis that terminates in the formation of sporangia, and the earliest cooksonioid vascular plant fossils reiterate this basic construction [1,2] (Figure 1). Later vascular plant fossils from the Rhynie chert assemblage have a variety of shoot architectures including indeterminate forms with lateral sporangia and leaves [1].

Vascular plant leaves have been classified into two types on the basis of morphological distinctions [1]. Whereas microphylls are small with a single vein, megaphylls are larger with complex venation patterns. However, leafless fossil precursors in lycophytes, monilophytes and seed plants show that leaves evolved independently in each vascular plant lineage (Figure 1B), and both microphylls and megaphylls have evolved by convergence in different groups [1,3].

The architectural innovations underpinning the radiation of vascular plant forms reflect differences in the structure and activities of meristems at the growing shoot tips. Whilst flowering plants have meristems that are multicellular with zones and layers with well characterised and specialised functions, monilophytes have meristems that comprise a single stem cell capping a more rapidly proliferative region (Figure 1C) [1]. Lycophyte meristems either have a single stem cell or a few stem cells depending on the group; again these overlie a more rapidly proliferative region [1].

With the exception of rhyniophytes, there is little fossil evidence of meristem structure at the bryophyte–vascular plant divergence [1]. It is therefore not yet clear whether there was a single or multiple evolutionary origins of meristematic indeterminacy in vascular plants. Amongst living bryophytes, only mosses have meristematic activities that resemble those of vascular plants. There is a transitory apical cell that iterates the embryo, and then the shoot axis is extended by the activity of a proliferative zone away from the tip termed the intercalary meristem [4]. Some liverworts and hornworts have proliferative regions that serve a similar function to the intercalary meristem of mosses [4] (Figure 1C).

The work reported by Frank *et al*. [5] aims to understand the molecular basis of evolutionary innovations in meristem function by identifying genes that regulate meristem function in species representing each major vascular plant lineage. Whereas reverse genetics with only a few key developmental gene families has previously been used to this end (reviewed in [3]), Frank *et al*. have used a wider transcriptomic approach [5].

Maize (*Zea mays*) was selected to represent seed plants, and *Equisetum* and *Selaginella* were selected to represent monilophytes and lycophyts, respectively. *Equisetum* and *Selaginella* both have meristems with apical cell(s) capping a proliferative zone. Although both have microphyllous leaves, they acquired leaves by convergence, and most monilophytes have megaphylls that evolved by convergence with seed plant leaves (Figure 1B) [1,3].

Frank *et al*. laser micro-dissected sectioned tissue from different meristem subdomains: the apical dome and P1 leaf primordium in maize, and the apical cell, the meristem core and P1 leaf primordia in *Equisetum* and *Selaginella* [5]. The transcriptomes of replica samples were Illumina sequenced and aligned to reference genomes, and the gene expression profiles of meristem zones within and between species were compared. Selected expression profiles were confirmed by in situ hybridization in *Selaginella*.

The paper finds that in the meristems of all three species there are distinct gene expression profiles in each apical domain sampled. This supports a model whereby, as in flowering plants, the meristems of *Selaginella* and *Equisetum* have functional zones comprising the apical stem cell(s), the meristem ‘core’– a proliferative zone subtending the apical cell(s)– and incipient leaves.

The developmental gene families expressed in each domain are largely distinct between *Selaginella* and *Equisetum*, supporting a model whereby the gene networks regulating meristem function by and large followed independent evolutionary trajectories in each lineage (Figure 1D). The *Equisetum* expression profile shares some overlap with maize but not *Selaginella*, indicating potential homologies in meristem function that evolved after the lycophyte–euphyllophyte divergence.

A smaller overlap between the expression profiles of *Selaginella* and maize, but not shared by *Equisetum*, could indicate either that *Selaginella* independently recruited similar genetic networks to regulate meristem function or that the networks regulating meristem function were originally shared between vascular plants, but then significantly modified in *Equisetum*.

The data presented are consistent with distinct patterns of evolution in each extant vascular plant lineage and the wide divergence time between lineages. Exceptions indicate potential genetic homologies in vascular plant meristem function and include *PIN*s, *DEK1*, and *LOG1*, which regulate auxin distributions [6], position dependent cell wall orientation [7] and the generation of active cytokinins [8] respectively. Intriguingly, disruption of *PIN* function in moss sporophytes can reproduce an architecture similar to *Partitatheca* fossils [6], indicating that roles for *PIN*s in driving meristem function may be conserved to bryophytes (Figure 1).

This spotlight positions the Frank *et al*. [5] paper in the context of the diversification of sporophytic shoot architectures because it is here that the data presented will be most informative. However, a similar transcriptomic approach extended to gametophytes could identify the genetic basis of convergence between generations, and determine how meristem function *per se* is constrained. A preliminary indicator is given by the necessity for *PINs*, *DEK1*, and cytokinin in driving gametophytic meristem functions in a moss (citations in [6,7]).

The broad nature of the Frank *et al*. [5] study pinpoints the relationship between the evolution of gene families, gene functions, and morphology as a significant unknown in our understanding of plant evolution, and suggests a high level of homoplasy. The advent of new high throughput sequencing initiatives (e.g., OneKP), and functional liverwort [9], hornwort [10], and monilophyte [11] models opens the exciting opportunity to identify the nature of this relationship and determine the molecular basis of morphological diversification during the colonisation of land.

## Figures and Tables

**Figure 1:**
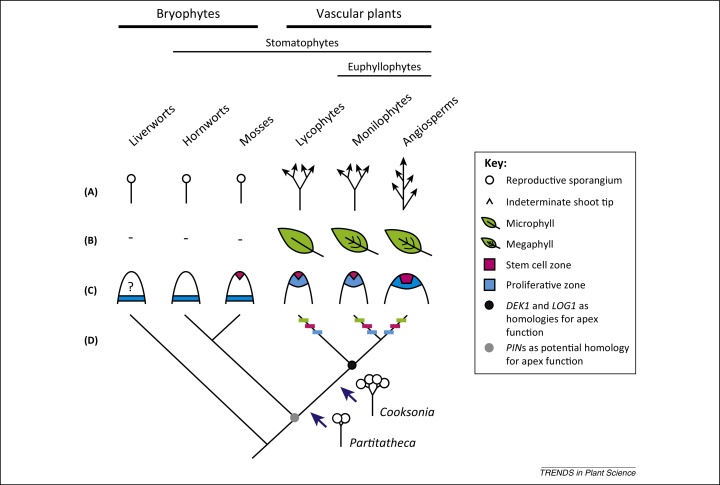
Evolutionary innovations underpinning the radiation of vascular plant shoot architectures. **(A)** Illustrates key transitions in overall shoot architecture at the bryophyte–vascular plant divergence. Whereas bryophytes have a single sporophytic axis that terminates in the formation of a reproductive sporangium, vascular plants have indeterminate shoots that branch by bifurcation (lycophytes and monilophytes) or have lateral branches (angiosperms) [1]. Fossil intermediaries between living bryophytes and vascular plants (e.g., *Partitatheca* and *Cooksonia*) have branching architectures with axes that terminate in sporangium formation [2]. **(B)** Illustrates the morphology of leaves that evolved independently in each vascular plant lineage. Whereas lycophytes typically have small leaves with a single vein (microphylls), monilophytes typically have larger leaves with complex venation patterns (megaphylls) [1]. The monilophyte sampled by Frank *et al*. [5] has microphylls, but evolved from megaphyllous ancestors (not shown). **(C)** Shows a general trend in land plant meristem morphology. Whereas angiosperm meristems have a multicellular stem cell zone (pink) surrounded by the more rapidly proliferative peripheral zone (blue), lycophytes and monilophytes have meristems with one to a few stem cells (pink) capping a proliferative zone (blue) [1]. Amongst bryophytes [4], only mosses share meristematic attributes with vascular plants. There is a transitory apical cell (pink) that makes the apical–basal axis, and this is extended by the activity of the intercalary meristem (blue). The sporangium is located between the two, and the juxtaposition of stem cell and proliferative zones during the evolution of vascular plants may have been a key switch permitting the evolution of indeterminate meristem function. **(D)** Hypotheses of sister relationship between bryophytes and vascular plants are currently in flux, but a recent phylotranscriptomic analysis suggested that liverworts comprise the earliest diverging lineage, and that hornworts and mosses jointly form a monophyletic sister group to vascular plants [12]. In conjunction with functional work in *Physcomitrella*[6] and this tree model, the new data from Frank *et al*. [5] place sporophytic PIN-regulated apex function as a potential homology of stomatophytes (grey spot), and DEK1 and LOG1-regulated apex function as a homology of vascular plants (black spot). Bars represent independent origins of leaves (green) and, as suggested by Frank *et al*. [5], independent recruitment of genetic networks to regulate stem cell function (pink) and proliferative functions (blue) in each vascular plant group.
